# Ultrasound Assessment of Carotid Plaque Echogenicity Response to Statin Therapy: A Systematic Review and Meta-Analysis

**DOI:** 10.3390/ijms160510734

**Published:** 2015-05-12

**Authors:** Pranvera Ibrahimi, Fisnik Jashari, Gani Bajraktari, Per Wester, Michael Y. Henein

**Affiliations:** Department of Public Health and Clinical Medicine, Umeå University, Umeå 901 87, Sweden; E-Mails: pranvera.ibrahimi@medicin.umu.se (P.I.); ganibajraktari@yahoo.co.uk (G.B.); per.wester@medicin.umu.se (P.W.); michael.henein@medicin.umu.se (M.Y.H.)

**Keywords:** carotid atherosclerosis, plaque echogenicity, ultrasound, statins

## Abstract

Objective: To evaluate in a systematic review and meta-analysis model the effect of statin therapy on carotid plaque echogenicity assessed by ultrasound. Methods: We have systematically searched electronic databases (PubMed, MEDLINE, EMBASE and Cochrane Center Register) up to April, 2015, for studies evaluating the effect of statins on plaque echogenicity. Two researchers independently determined the eligibility of studies evaluating the effect of statin therapy on carotid plaque echogenicity that used ultrasound and grey scale median (GSM) or integrated back scatter (IBS). Results: Nine out of 580 identified studies including 566 patients’ carotid artery data were meta-analyzed for a mean follow up of 7.2 months. A consistent increase in the echogenicity of carotid artery plaques, after statin therapy, was reported. Pooled weighted mean difference % (WMD) on plaque echogenicity after statin therapy was 29% (95% CI 22%–36%), *p <* 0.001, I^2^ = 92.1%. In a meta-regression analysis using % mean changes of LDL, HDL and hsCRP as moderators, it was shown that the effects of statins on plaque echogenicity were related to changes in hsCRP, but not to LDL and HDL changes from the baseline. The effect of statins on the plaque was progressive; it showed significance after the first month of treatment, and the echogenicity continued to increase in the following six and 12 months. Conclusions: Statin therapy is associated with a favorable increase of carotid plaque echogenicity. This effect seems to be dependent on the period of treatment and hsCRP change from the baseline, independent of changes in LDL and HDL.

## 1. Introduction

Carotid atherosclerosis is an important cause of ischemic stroke, the risk of which is mainly related to the degree of stenosis [1]. Adding the evaluation of carotid plaque echogenicity features, on the other hand, it was found to better risk stratify patients beyond the degree of stenosis. Treatment of carotid atherosclerosis with statins has proven effective in reducing such stroke risk, universally considered to be caused by vulnerable plaques [2].

Many imaging techniques are currently used to identify vulnerable plaque features. The most feasible one with less radiation remains carotid ultrasound, which can accurately identify the presence of the plaque and determine its echogenicity, as well as the degree of stenosis. Vulnerable plaques are known for their high lipid and hemorrhage content, in contrast to stable plaques, which are predominately rich in fibrous tissue and calcification [3,4]. Furthermore, such detailed plaque composition has been found to correlate with the textural features (echogenicity) obtained by ultrasound imaging. This can easily be assessed using off-line plaque image analysis techniques, such as grey scale median (GSM) and integrated backscatter (IBS), with plaques rich in lipid and hemorrhagic content appearing echolucent (low GSM or IBS) and those with fibrous or calcific content appearing echogenic (high GSM or IBS) [5,6].

The effect of statins treatment on plaque regression and change in its features is well documented in the literature, but a consensus analysis is lacking. Such an effect has been reported using various imaging modalities, other than US [7], not only in carotid disease, but also in coronary and aortic disease [8]. The aim of this study was to determine, in a systematic and meta-analysis model, the response of plaque features’ “echogenicity” to statin therapy in patients with carotid artery disease.

## 2. Results

### 2.1. Study Selection

**Figure 1:**
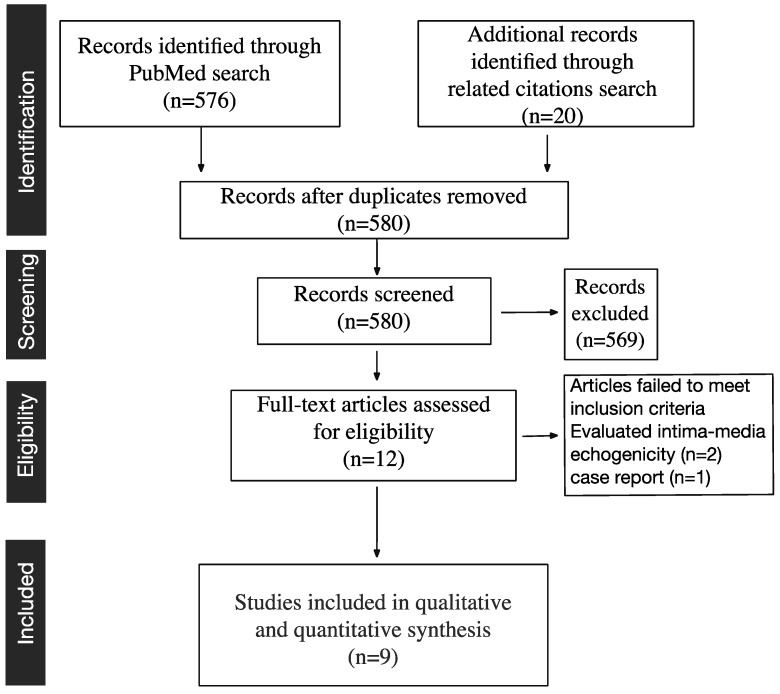
Study selection.

We identified 576 studies in total after systematic searching in PubMed (Figure 1). No additional studies were found in MEDLINE, EMBASE or in the Cochrane Center Register. After reading the titles and abstracts of the papers, we first depicted 12 studies that evaluated the effects of statins on plaque echogenicity. Of these studies, three were excluded, two measured the effect of statins on the carotid artery wall (intima-media) echogenicity [9,10] and one was a case report [11]. The remaining nine studies [12,13,14,15,16,17,18,19,20] were included in the final qualitative analysis (Figure 1, Table 1 and Table 2). Out of the nine studies, two studies analyzed two groups of patients separately, and we have included both groups in the meta-analysis. In addition, we have used meta-regression and subgroup analysis to determine the effect of % changes in LDL, HDL, hsCRP, the period of treatment and baseline patients’ characteristics on echogenicity change. Statins effect on LDL, HDL and hsCRP were also evaluated (Supplementary Material).

### 2.2. Qualitative Assessment and Study Characteristics

#### 2.2.1. Effect of Statins on Plaque Features

All nine studies included were prospective; five were prospective open-label, and four of them were randomized controlled trials [8,9,10,11]. Atorvastatin was the most commonly used [13,15,17,18], followed by simvastatin [13], pravastatin and pitavastatin [12,14] and rosuvastatin [20]. In three studies, the statin dosage was fixed [14,16,19], and in the remaining six, it was ranged [12,13,15,17,18,20]. The mean follow up of patients was 7.2 months (1–12). Studies included in this review used different methods to quantify plaque echogenicity. The grey scale median (GSM) was used in five studies to evaluate plaque echogenicity, and integrated backscatter (IBS) analysis was used in the remaining four. In one study, patients were divided into two groups based on the statin dose (intensive *vs.* moderate) [18] and, in the other one, based on treatment strategy (statins + carotid artery stenting (CAS) *vs.* statin alone) [17]. In seven studies [12,13,14,16,17,18,20], controls were commenced on a set diet, and in one study [15], patients on statins were compared with those on placebo. The diet type applied in the controls was mentioned in two studies [12,19], which used the Adult Treatment Panel-III lipid-lowering diet. There was only one study that used more than one type of statin in the treated group [13].

In all nine studies presented in this meta-analysis, there was a significant increase of plaque echogenicity after statin therapy. In the compared high (atorvastatin 80 mg/d) and low (atorvastatin 20 mg/d) statin therapy, the GSM was significantly increased more in the group receiving aggressive statin therapy.

In six studies, other ultrasound-derived measurements were evaluated, including: intima-media thickness [12,16,20], plaque thickness [13,19], plaque volume [16] and degree of stenosis [12]. Except for one study that found a decrease in plaque thickness after statin therapy [13], there was no other change, of the above-mentioned measures, observed after statin therapy.

#### 2.2.2. Effect of Statins on Blood Lipids and Inflammatory Markers

All studies measured LDL and HDL cholesterol levels, and in all, there was a significant decrease in LDL with statin therapy; in only two [12,14] did the HDL cholesterol significantly increase. However, only one study has evaluated the change of plaque echogenicity after adjusting for LDL cholesterol and its changes from baseline, which concluded that statins’ effect on plaque echogenicity was independent of the fall in LDL cholesterol [19].

The included studies used different blood-derived markers of atherosclerosis, such as: high sensitivity CRP (hsCRP) [12,13,14,15,16,17,18,20], vasogenic endothelial growth factor (VEGF) [14], interleukins (IL) IL-6 and IL-18 [13], osteopontin (OPN) [15,17,18], osteoprotegerin (OPG) [15,17,18] and tumor necrosis factor-alpha (TNF-α) [14]. Except for IL-6, which was not affected [13], the other markers, hsCRP, VEGF, IL-18, OPN, OPG and TNF-α, were significantly decreased in patients treated with statins compared to controls. Even at only one month after statin therapy, the levels of hsCRP, VEGF and TNF-α decreased significantly [14]. In the study that compared aggressive (atorvastatin 80 mg/d) and modest (atorvastatin 10 mg/d) statin therapy, OPN and OPG levels were lower in patients receiving aggressive statin therapy; however, hsCRP was not different between groups [18].

**Table 1:** Studies included in meta-analysis.

Author/Year	Population (*n*)	Mean Age ± SD	Gender (Male)	Hypercholesterolemic	Carotid Stenosis	Echogenicity Measured	Minor Score
1. Watanabe *et al.*, 2005 [12]	30	69.9 ± 8.8	63%	No	Moderate	IBS	RT
2. Yamagami *et al.*, 2008 [13]	41	63.4 ± 8.3	24%	Yes	Moderate	IBS	RT
3. Nakamura *et al.*, 2008 [14]	33	60 ± 9	25%	Yes	Moderate	IBS	RT
4. Kadoglou *et al.*, 2008 [15]	113	63.6 ± 9.9	67%	Yes	Moderate symptomatic	GSM	20
5. Yamada *et al.*, 2009 [16]	40	71 ± 8	90%	No	30%–60%	IBS	RT
6. Kadoglou *et al.*, 2009 [17]	67 + 46	66.7 ± 7.3	40%	No	>40%	GSM	20
7. Kadoglou *et al.*, 2010 [18]	66 + 65	64.9 ± 10	46%	Yes	30%–60%	GSM	24
8. Della-Morte *et al.*, 2011 [19]	40	>45	NA	Yes	NA	GSM	15
9. Nohara *et al.*, 2013 [20]	25	63.9 ± 8.1	50%	Yes	NA	GSM	20

GSM, grey scale median; IBS, integrated back scatter; RT, randomized trial; SD, standard deviation.

**Table 2:** Studies characteristics and statins effect on plaque echogenicity and LDL, HDL and hsCRP level on the blood.

Author/Year	Study Design	Statin/Dose	Follow-up (Months)	% Change Echogenicity	% Change LDL	% Change HDL	% Change hsCRP
1. Watanabe *et al.*, 2005 [12]	Randomized case-control trial	Pravastatin	6	14.1 ± 3.3	24.5 ± 6.4	10.2 ± 6.0	45.0 ± 58.3
3. Yamagami *et al.*, 2008 [13]	Randomized case-control trial	Simvastatin 10 mg	1	10.6 ± 4.3	34.2 ± 18.4	0	43.0 ± 119.8
2. Nakamura *et al.*, 2008 [14]	Randomized case-control trial	Pitavastatin 4 mg	12	32.1 ± 5.9	37.8 ± 12.4	9.3 ± 2.0	43.7 ± 51.5
4. Kadoglou *et al.*, 2008 [15]	Open-label prospective trial	Atorvastatin	6	36.0 ± 15.2	41.7 ± 19.9	4.5 ± 2.4	58.9 ± 34.0
5. Yamada *et al.*, 2009 [16]	Randomized case-control trial	Simvastatin	6	17.0 ± 5.9	44.0 ± 23.9	0	42.1 ± 94.6
6a. Kadoglou *et al.*, 2009 [17]	Open-label prospective trial	Atorvastatin	6	36.8 ± 9.8	38.6 ± 20.0	13.4 ± 6.6	78.3 ± 74.9
6b. Kadoglou *et al.*, 2009 [17]	Open-label prospective trial	Atorvastatin + CAS	6	48.4 ± 18.6	33.3 ± 15.0	4.4 ± 2.2	52.1 ± 39.5
7a. Kadoglou *et al.*, 2010 [18]	Randomized case-control trial	Atorvastatin 10–20 mg	12	32.6 ± 11.7	64.5 ± 23.6	5.5 ± 2.6	52.9 ± 55.2
7b. Kadoglou *et al.*, 2010 [18]	Randomized case-control trial	Atorvastatin 80 mg	12	51.4 ± 18.4	54.2 ± 37.2	10.3 ± 6.0	65.0 ± 80.0
8. Della-Morte *et al.*, 2011 [19]	Prospective pilot study	NA	1	21.9 ± 4.8	51.4 ± 31.0	2.0 ± 1.1	NA
9. Nohara *et al.*, 2013 [20]	Prospective open label, blinded-endpoint	Rosuvastatin	12	16.9 ± 33.1	50.1 ± 22.9	8.1 ± 3.6	NA

CAS, carotid artery stenting.

### 2.3. Meta-Analysis Results

**Figure 2:**
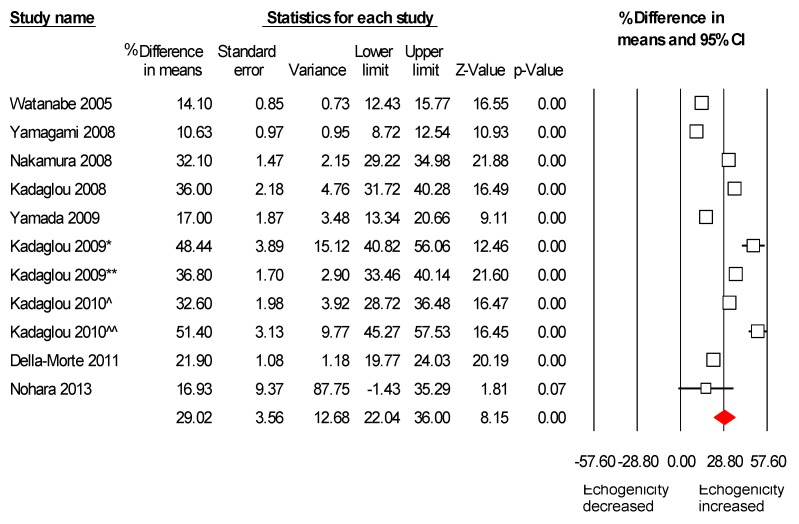
Effects of statin on plaque echogenicity. Note: Kadaglou 2009 [17] has divided and analyzed patients in two groups: the first group (*) was on statin, but underwent contralateral carotid artery stenting (CAS), and the second group (**) was treated only with statins. Kadaglou 2010 [18] has divided and analyzed patients into two groups: the first group (^) received atorvastatin 10–20 mg, and the second group (^^) atorvastatin 80 mg.

All nine studies met the inclusion criteria to be included in the meta-analysis. Two of the studies have divided patients into two groups. The first one [18] had two groups on different statin dosage (atorvastatin 10 *vs.* 80 mg), and the other study [17] had two groups on statin therapy; one of them underwent CAS in addition. In total, 566 patients’ carotid artery data were meta-analyzed for a mean follow up of 7.2 months. A consistent increase in the echogenicity of carotid artery plaques, after statin therapy, was found. Pooled weighted mean difference % (WMD) on plaque echogenicity after statin therapy was 29% (95% CI: 22%–36%), *p <* 0.001, I^2^ = 92.1% (Figure 2). In these studies, evaluating the effect of statins on plaque echogenicity was the main objective; in addition, their effect on LDL, HDL and hsCRP level was also evaluated. LDL was significantly decreased after statins therapy; the pooled weighted mean difference % (WMD) was −40.8% (95% CI, −48.9%–−32.8%), *p <* 0.001, I^2^ = 81.2% (Supplemental Figure S1). Furthermore, HDL and hsCRP were increased and decreased after statin therapy, respectively (Supplemental Figures S2 and S3).

**Figure 3:**
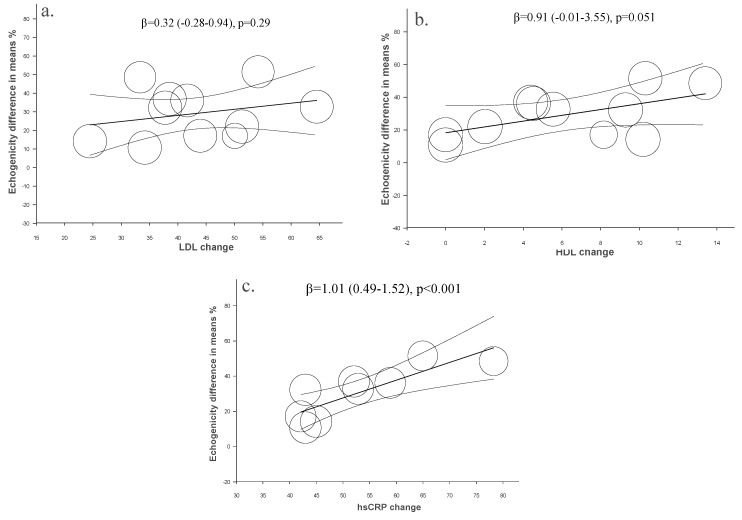
Meta-regression. Regression of LDL (**a**), HDL (**b**) and hsCRP (**c**) changes on plaque echogenicity after statin therapy.

In a meta-regression analysis, mean changes % of LDL, HDL and hsCRP from baseline were used as moderators to evaluate their association with changes in plaque echogenicity. The increase in plaque echogenicity with statins therapy was independent of LDL (β = 0.32 (−0.28–0.94), *p <* 0.29) (Figure 3a) and HDL cholesterol (β = 0.91 (−0.01–3.55), *p* = 0.051) (Figure 3b), but it was related to hsCRP changes from the baseline (β = 1.01 (0.49–1.52), *p <* 0.001) (Figure 3c).

**Figure 4:**
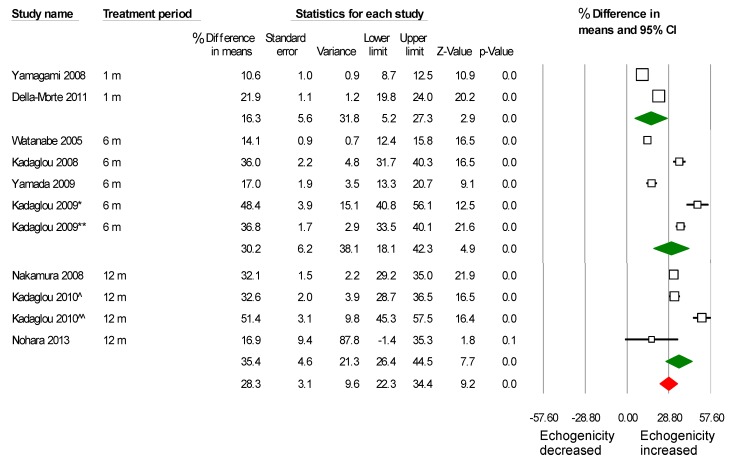
Analysis of studies based on treatment period in months. The effect of statins on plaque echogenicity was obvious from the first month after treatment, and the effect was progressive on the following six and 12 months (m). (*) was on statin, but underwent contralateral carotid artery stenting (CAS), and (**) was treated only with statins. (^) received atorvastatin 10–20 mg, and (^^) atorvastatin 80 mg.

**Figure 5:**
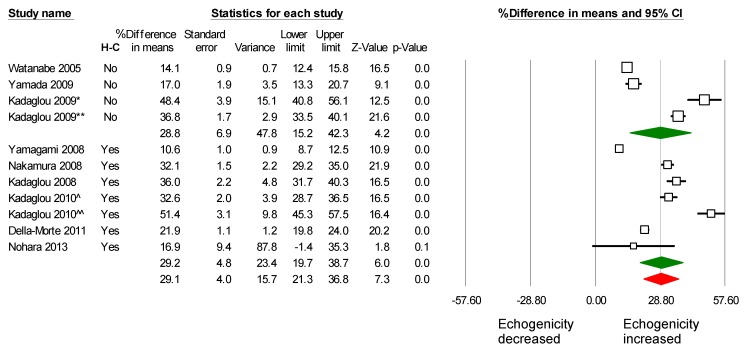
The effect of statins on plaque echogenicity was similar in hypercholesterolemic and non-hypercholesterolemic patients. H–C = hypercholesterolemia at baseline. (*) was on statin, but underwent contralateral carotid artery stenting (CAS), and (**) was treated only with statins. (^) received atorvastatin 10–20 mg, and (^^) atorvastatin 80 mg.

Patients were divided into subgroups and analyzed based on treatment period. Although the increase of plaque echogenicity was significant even after the first month of treatment, this increase was more evident in the following six and 12 months (Figure 4). Mean difference % was 16.2% (5.2%–27.2%) *vs.* 30.4% (18.2%–42.3%) *vs.* 35.4% (26.3%–44.4%), *p* = 0.03, between 1, 6 and 12 months of treatment, respectively. In addition, we have analyzed separately studies based on baseline cholesterolemia status (hypercholesterolemic *vs.* non-hypercholesterolemic), and it seems that the effects of statins on carotid plaque echogenicity is independent of cholesterol levels at baseline, since it was similarly increased in both groups (Figure 5).

### 2.4. Assessment of Potential Publication Bias

No publication bias was noted in our analyses (Table 3).

**Table 3:** Heterogeneity and publication bias measures.

	Test of Heterogeneity	Publication Bias (Begg and Mazumdar Rank Correlation)		
	I^2^	Kendell Tau	Test Statistic Z	*p*-Value
Echogenicity	92.1	0.34	1.41	0.16
LDL	81.2	−0.33	1.25	0.12
HDL	98.1	0.05	0.20	0.41
hsCRP	0	0	0	1.0

## 3. Discussion

Atherosclerosis is a long-lasting pathology with well-established stages starting with mild wall thickness and ending with complete fibrosis and calcification [1]. Along the course of the disease, plaques are formed mainly based on a lipid core, and they too are subject to structural and functional changes over time [21]. Increasing plaque area and volume while parts of it might be healing and developing fibrosis and spotty calcification characterize active plaque pathology. Soft plaques can easily be identified by various imaging techniques, including ultrasound, which has shown that echolucent plaques are the ones associated with potential complications [22,23], including even micro-emboli [24] and strokes [25].

Statins are well-established treatment for atherosclerosis and its complications. Their beneficial clinical effect, in the form of reduced events, e.g., stroke and coronary syndromes, is through lowering LDL-cholesterol levels [2] and their anti-inflammatory effect [26]; the two mechanisms result in volume reduction and plaque stabilization, as shown by increased plaque echogenicity. Our results support that pathway; however, in addition, they show that the increase in plaque echogenicity seem to be independent of the changes in intima-media thickness, plaque area or volume. These findings suggest that the statins-related increase in plaque echogenicity represents an early effect that could be used for monitoring individual patient’s response to therapy. Indeed, evidence exists that the effect of statins on plaque volume appears later, after changes in echogenicity. This is not a unique feature of just carotid disease, but also coronary plaques, which have been shown to demonstrate quantitative regression in volume after 19 months of statins therapy [8]. In our meta-regression analysis, the effect of statins on plaque echogenicity was also independent of changes in LDL and HDL levels, but was related to changes of hsCRP levels. In addition, the increased echogenicity was higher in patients treated for a longer period, again irrespective of the cholesterol level at baseline.

Current data indicate that higher statins doses (atorvastatin 80 mg) have a more potent effect on increasing plaque echogenicity compared to smaller doses (atorvastatin 20 mg). These findings mirror those we previously showed in coronary artery disease, with higher statins doses resulting in a faster rate of coronary calcification compared to smaller doses [27]. Furthermore, the effect of statins duration on plaque features mirrors what we recently reported in the coronary circulation [28].

In addition to the beneficial clinical effect of statins on plaque features and potential stability, our analysis shows that ultrasound carotid imaging plays a pivotal role in early and potential continuous monitoring of such an effect. Atherosclerotic plaques can be detected and their features studied by CT and MRI scanning; however, the two techniques are known for their significant limitations, particularly radiation in the former and claustrophobia in the latter, adding to their higher cost compared with ultrasound. With carotid ultrasound free of those limitations, its accuracy in studying plaque echogenicity makes it emerge as a unique non-invasive image modality ideal for early identification of the disease and accurate monitoring of its progress and response to treatment. Ultrasound, on the other hand, has the limitation of being more time consuming with a potential inter-observer variability, particularly so when using plaque characterization methods that are, to some extent, superior to the conventionally-used intima-media thickness for early disease detection [29]. Finally, statins are usually well-tolerated medications with few adverse effects, including myopathy and elevation of liver enzymes. Rhabdomyolysis is a rare related complication [30]. Likewise, liver dysfunction is very rare, which was a reason for the FDA to remove the old recommendation of routine monitoring of liver enzymes [31]. As for diabetes mellitus, two comprehensive meta-analyses [32,33] have shown a slightly increased risk of diabetes development in subjects on statin therapy; however, the risk is low both in absolute terms and when compared with the reduction in cardiovascular events.

Study limitations: A systematic review based on relevant key words might have missed some relevant publications, but the search was checked by two investigators blinded to each other’s means of search. We used only publications in the English language; other relevant ones in different languages might have been missed. Another limitation was the low number of patients included in studies and that different studies have used different statins of variable dosages. Patients were followed up for different periods of time, and in most studies, statins dosage was ranged, thus limiting us to assessing the dose effect on plaque echogenicity using the meta-regression analysis. This is the nature of searching various studies of different designs.

Clinical implications: Our results support the use of carotid ultrasound analysis of plaque features and echogenicity as a marker of plaque stability in response to statins therapy. These changes are independent of plaque area or volume, suggesting that they might reflect an early effect before anatomical response and plaque shrinking is detected. In addition, the effects of statins on the plaque were progressive and independent of baseline cholesterol levels. Applying this method in monitoring individuals at high risk for vascular events might support treatment adjustment for targeting better clinical outcome.

## 4. Experimental Section

### 4.1. Methods

The methodology for this study was based on the Preferred Reporting Items for Systematic Reviews and Meta-Analyses statement [34].

### 4.2. Information Search and Data Collection

Up to April, 2015, we systematically searched electronic databases (PubMed, MEDLINE, EMBASE and Cochrane Center Register) for studies evaluating the effect of statins on carotid plaque echogenicity. The search terms used were: “carotid atherosclerosis”, “carotid plaque”, “ultrasound” “statins”, “HMG-CoA reductase inhibitors” and “lipid-lowering drugs”, in various combinations. Two researchers (Pranvera Ibrahimi and Fisnik Jashari), independent of each other, performed the literature search, study selection and data extraction. There was no time, language or publication limit in the literature search. The selected reports were manually searched, and relevant publications, obtained from the reference lists, were retrieved.

### 4.3. Study Eligibility Criteria

Clinical studies that reported results on the effect of statin therapy on the plaque echogenicity (GSM, IBS) evaluated by duplex ultrasound were eligible. Specific inclusion criteria were: (1) observational, non-randomized or randomized studies that explored the effect on statin treatment either as primary or secondary cardiovascular disease prevention; (2) ultrasound of the carotid arteries before and at least once at a follow-up of at least one month; (3) English language articles; (4) studies with ≥15 subjects; and (5) ultrasound-based characterization of carotid artery plaque composition. All other studies that used different imaging techniques (e.g., MRI, CT, IVUS, PET) and those that used plaque features other than echogenicity (volume, degree of stenosis, ulceration, neovascularization) as a target for monitoring statin therapy were excluded. We have performed a quality score of the retrieved studies utilizing the methodological index for the non-randomized studies (MINORS) [35]. Studies that scored over 20 out of 24 (or 14 out of the 16 for those non-comparative, but rather solely observational) were considered of adequate quality. In the meta-analysis, we included studies that specified duration of the study and presented plaque echogenicity means and standard deviations prior to and during (or at the completion of) the intervention or the percent change in plaque echogenicity before and during intervention.

### 4.4. Statistical Analyses

For the plaque echogenicity analysis, the treatment effects of interest were the differences in the extent of changes in echogenicity (GSM or IBS), low-density lipoprotein cholesterol (LDL), high-density lipoprotein (HDL) and high sensitivity C-reactive protein (hsCRP) before and after treatment. Because of the significant variation in study size, length and follow-up, as well as patient’s characteristics, we have used random-effects. Heterogeneity was measured using I^2^ statistics. We performed analyses within each imaging group stratified by pre- *vs.* post-treatment. All analyses were conducted using Comprehensive Meta Analysis Version 3 software (Biostat inc., Englewood, NJ, USA).

## 5. Conclusions

Statins therapy is associated with a favorable increase of carotid plaque echogenicity, even after one month of treatment. This effect is independent of changes in plaque morphology and, furthermore, is more profound using higher doses of statins. Finally, the effect of statins seems to be related to the decrease of hsCRP from the baseline rather than dyslipidemia.

## Supplementary Materials

Supplementary materials can be found at http://www.mdpi.com/1422-0067/16/05/10734/s1.
