# Supplementary material for: Ultrasound Assessment of Carotid Plaque Echogenicity Response to Statin Therapy: A Systematic Review and Meta-Analysis
Source: Int J Mol Sci. 2015 May 12;16(5):10734–47. doi: 10.3390/ijms160510734 (PMC4463673; doi:10.3390/ijms160510734)
Supplement: Supplementary file 1 [file ijms-16-10734-s001.pdf]

Supplementary Information

Study name	% Difference in means	Standard error	Variance	Lower limit	Upper limit	Z-Value	p-Value
Watanabe 2005	-24.50	1.56	2.43	-27.56	-21.44	-15.71	0.00
Yamagami 2008	-34.20	4.11	16.93	-42.26	-26.14	-8.31	0.00
Nakamura 2008	-37.80	3.05	9.32	-43.78	-31.82	-12.38	0.00
Kadaglou 2008	-41.70	2.86	8.17	-47.30	-36.10	-14.59	0.00
Yamada 2009	-44.00	7.56	57.12	-58.81	-29.19	-5.82	0.00
Kadaglou 2009*	-33.36	3.14	9.86	-39.51	-27.21	-10.62	0.00
Kadaglou 2009**	-38.60	3.46	11.94	-45.37	-31.83	-11.17	0.00
Kadaglou 2010^	-64.56	4.34	18.80	-73.06	-56.06	-14.89	0.00
Kadaglou 2010^^	-54.20	6.29	39.54	-66.52	-41.88	-8.62	0.00
	-40.88	4.11	16.87	-48.93	-32.83	-9.95	0.00

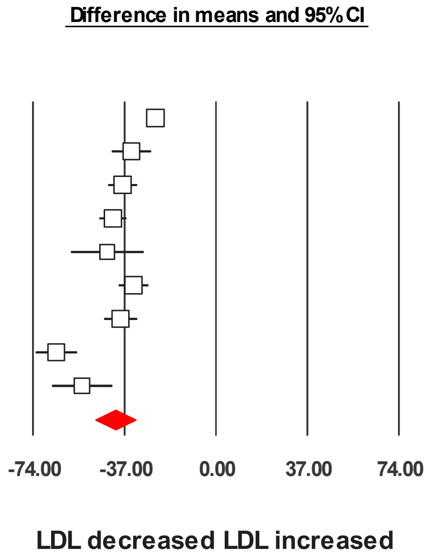

Figure S1. Effect of statins on LDL. Note: Kadaglou 2009 [17] has divided and analyzed patients into two groups: the first group (*) was on statin, but underwent contralateral carotid artery stenting (CAS), and the second group (**) was treated only with statins. Kadaglou 2010 [18] has divided and analyzed patients into two groups: the first group (^) received atorvastatin 10–20 mg and the second group (^^) atorvastatin 80 mg.

Study name	% Difference in means	Standard error	Variance	Lower limit	Upper limit	Z-Value	p-Value
Watanabe 2005	10.20	1.56	2.43	7.14	13.26	6.54	0.00
Yamagami 2008	0.00	0.04	0.00	-0.09	0.09	0.00	1.00
Nakamura 2008	9.30	0.49	0.24	8.33	10.27	18.89	0.00
Kadaglou 2008	4.54	0.35	0.12	3.85	5.23	12.85	0.00
Yamada 2009	0.00	0.06	0.00	-0.12	0.12	0.00	1.00
Kadaglou 2009*	13.42	1.38	1.89	10.72	16.12	9.75	0.00
Kadaglou 2009**	4.40	0.39	0.15	3.64	5.16	11.42	0.00
Kadaglou 2010^	5.56	0.44	0.19	4.70	6.42	12.60	0.00
Kadaglou 2010^^	10.32	1.02	1.04	8.33	12.31	10.14	0.00
	5.66	0.60	0.36	4.49	6.83	9.47	0.00

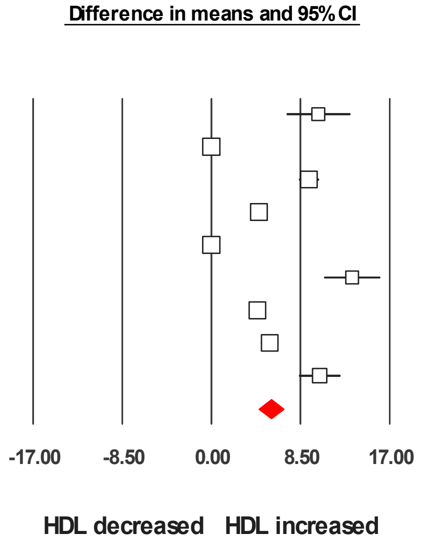

Figure S2. Effects of statins on HDL. Note: Kadaglou 2009 [17] has divided and analyzed patients into two groups: the first group (*) was on statin, but underwent contralateral carotid artery stenting (CAS), and the second group (**) was treated only with statins. Kadaglou 2010 [18] has divided and analyzed patients into two groups: the first group (^) received atorvastatin 10–20 mg and the second group (^^) atorvastatin 80 mg.

Study name	Statistics for each study						
	% Difference in means	Standard error	Variance	Lower limit	Upper limit	Z-Value	p-Value
Watanabe 2005	-45.00	15.06	226.83	-74.52	-15.48	-2.99	0.00
Yamagami 2008	-43.00	26.79	717.60	-95.50	9.50	-1.61	0.11
Nakamura 2008	-43.70	12.68	160.74	-68.55	-18.85	-3.45	0.00
Kadaglou 2008	-58.90	4.88	23.84	-68.47	-49.33	-12.06	0.00
Yamada 2009	-42.10	29.92	894.92	-100.73	16.53	-1.41	0.16
Kadaglou 2009*	-78.30	15.62	243.91	-108.91	-47.69	-5.01	0.00
Kadaglou 2009**	-52.10	6.82	46.57	-65.48	-38.72	-7.63	0.00
Kadaglou 2010^	-52.90	9.34	87.28	-71.21	-34.59	-5.66	0.00
Kadaglou 2010^^	-65.00	13.52	182.86	-91.50	-38.50	-4.81	0.00
	-55.84	3.20	10.25	-62.11	-49.57	-17.44	0.00

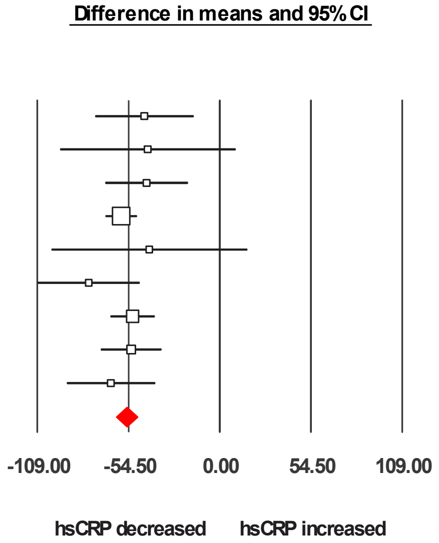

Figure S3. Effects of statins on hsCRP. Note: Kadaglou 2009 [17] has divided and analyzed patients into two groups: the first group (*) was on statin, but underwent contralateral carotid artery stenting (CAS), and the second group (**) was treated only with statins. Kadaglou 2010 [18] has divided and analyzed patients into two groups: the first group (^) received atorvastatin 10–20 mg and the second group (^^) atorvastatin 80 mg.
